# Safety of rapid intravenous paracetamol infusion in paediatric patients

**DOI:** 10.1016/j.crphar.2021.100077

**Published:** 2021-12-18

**Authors:** Astrid Eliasen, Sigrid Otnes, Merete Matz, Lise Aunsholt, René Mathiasen

**Affiliations:** a Department of Clinical Pharmacology, Bispebjerg and Frederiksberg University Hospital, Bispebjerg Bakke 23, indgang 20C, 2. sal, 2400, Copenhagen, Denmark; b Department of Pediatrics and Adolescent Medicine, Copenhagen University Hospital Rigshospitalet, Blegdamsvej 9, 2100, Copenhagen, Denmark; c Institute of Clinical Medicine, Faculty of Medicine, University of Copenhagen, Blegdamsvej 3B, 2200, Copenhagen, Denmark; d Department of Neonatal and Pediatric Intensive Care, Copenhagen University Hospital Rigshospitalet, Blegdamsvej 9, 2100, Copenhagen, Denmark

**Keywords:** Paracetamol, Acetaminophen, Children, Adolescents, Paediatrics, Neonates

## Abstract

**Purpose:**

Paracetamol is recommended as a first-line treatment for pain and fever in paediatric patients. Intravenous (IV) infusions are recommended to be administered as a 15-min infusion to minimize local tissue trauma and related pain. The purpose of this study was to demonstrate that IV paracetamol could be administered during 5 ​min or less in paediatric patients without causing related adverse reactions.

**Methods:**

Prospective, observational safety study including children aged <18 years who received IV paracetamol. Pain scores before and after the paracetamol infusions were obtained using VAS, FLACC, COMFORT neo, or COMFORT behaviour scales with scores from 0 to 10 representing no pain to worst pain. Further, objective signs of inflammation at the infusion site were registered.

**Findings:**

We included 44 patients (median age 2.8 years, range 0.01–17.0 years) who received paracetamol in a peripheral venous catheter (n ​= ​22) or central venous catheter (n ​= ​22). In total, the 93 paracetamol infusions had a median infusion time of 3:00 ​min, range 0:40 to 5:00 ​min. After infusions, pain scores were lower, compared to before infusions (mean change −0.26, 95% confidence interval −0.45 to −0.07, *P* ​= ​0.007), and no objective signs of inflammation were reported.

**Implications:**

This safety study indicates that IV paracetamol can be administered in paediatric patients with a shorter infusion time than recommended without causing adverse reactions. The results may contribute to a more efficient workflow at paediatric departments.

## Highlights

• Paracetamol is widely used for treating pain and fever in paediatric patients.
• The recommended infusion time of intravenous paracetamol is 15 ​min.
• The infusion time can safely be reduced to ≤5 ​min in paediatric patients.

## Graphical abstract

**Figure:**
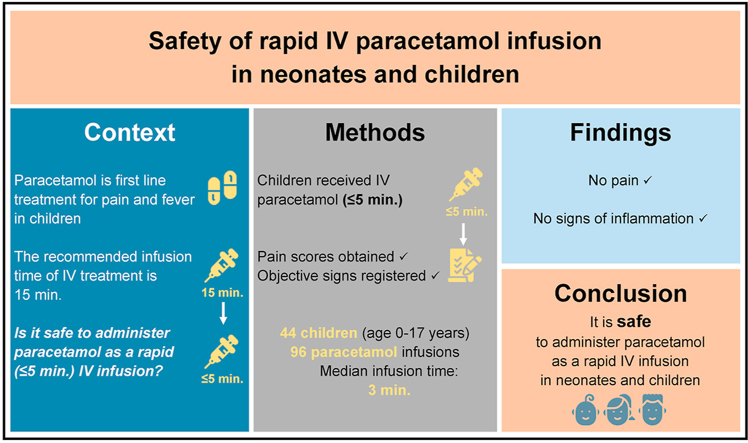

## 1 Introduction

Paracetamol (acetaminophen) is widely used to treat pain and reduce fever in children (Meremikwu and Oyo-Ita, 2002; Perrott et al., 2004). With a favourable therapeutic profile, including a good safety record at recommended doses, no significant drug–drug interactions, and multiple administration forms, this drug is suitable for use in children at any age (Perrott et al., 2004; Tan et al., 2020). However, due to variation in the bioavailability after oral and rectal administrations, the onset and duration of analgesia may be slow and unpredictable (Sharma and Mehta, 2014). Meanwhile, intravenous (IV) administration ensures analgesia within 5 ​min and is therefore preferred when immediate pain relief is needed or in situations where oral or rectal administration is not possible.

Paracetamol is unstable in solutions and poorly soluble in water; therefore, propacetamol was introduced in the 1980s. Propacetamol is a water-soluble prodrug of paracetamol developed for parenteral administration, but the unphysiological osmolarity and pH value may induce pain during infusion. When prolonged from 2 to 15 ​min, this infusion-related pain could be reduced (Depré et al., 1992). In the early 2000s, an IV formulation of paracetamol was approved as a ready-to-use solution with physicochemical properties closer to plasma and a significantly reduced risk of local infusion-related adverse reactions compared to propacetamol (2% vs. 38%, p ​< ​0.001) (Flouvat et al., 2004; Sinatra et al., 2005). However, the duration of infusion of paracetamol was still advised to be 15 ​min. A retrospective study has shown that IV paracetamol can be administered as a rapid infusion in adults with a duration of 4 ​min or less without causing adverse reactions (Needleman, 2013). We hypothesized that paracetamol could be administered intravenously with a duration of infusion of 5 ​min or less in children aged <18 years without causing subjective or objective infusion-related adverse reactions. To test our hypothesis, we compared the mean pain score before and after paracetamol infusion in paediatric patients and registered signs of inflammation at the infusion site.

## 2 Methods

This prospective, observational safety study was conducted at the Department of Paediatric Oncology, the Semi-intensive Paediatric Unit, and the Department of Neonatal and Paediatric Intensive Care at Copenhagen University Hospital Rigshospitalet in Denmark between October 29, 2020, and May 19, 2021. The study protocol was approved by the institutional review board, and approval for administering IV paracetamol with an infusion time of 5 ​min or less was obtained as a change in practice before the study began.

### 2.1 Patients and treatment

Children aged <18 years who received paracetamol as an IV infusion via a peripheral or central venous catheter (PVC, CVC) were eligible for inclusion. Nurses were instructed to administer paracetamol with a duration of infusion of 5 ​min or less. Patients were excluded if the duration of infusions were more than 5 ​min. The dosing schedule of paracetamol was three to four times daily using weight-based doses for paediatric patients and doses based on gestational age for neonatal patients. Paracetamol was administered using both unopened and reused vials. Paracetamol was administered manually via a syringe.

### 2.2 Assessment of adverse reactions and study outcomes

The treating nurses registered pain intensity scores before and during or immediately after the paracetamol infusion. Validated pain scales, corresponding to age and cognitive development of the child, were used: Visual Analogue Scale (VAS), Face, Legs, Activity, Cry, Consolability (FLACC), COMFORT neo, or COMFORT behaviour. For the VAS and FLACC scales, scores between 0 and 10 represented no pain to worst pain. For the COMFORT scales, scores between 6 and 30 represented levels of distress, while scores between 0 and 10 represented pain. Local pain was registered when infusions were administered via a PVC, and general pain was registered when infusions were administered via a CVC. Further, objective signs of inflammation (redness, swelling, heat) at the infusion site were registered for PVC administrations. Data were collected using a web-based survey developed in REDCap (Harris et al., 2019). For each patient, an electronic case report form was completed including the Danish Personal Identification number (CPR), primary diagnosis, indication for paracetamol prescription, date, infusion time (minutes and seconds), catheter type, pain assessment method, pain score before and after infusion, and objective signs of inflammation (for PVC). It was optional to state free-text comments.

The main outcome was a change in mean pain score before and after paracetamol infusion.

We aimed to include 40 consecutive patients and allowed more than one data registration on individual patients.

Statistical analyses were performed using R version 3.6.1. Two-tailed paired *t* tests were used to test for differences in mean pain score before and after paracetamol infusion with a *P* value ​< ​0.05 considered statistically significant.

## 3 Results

We included 44 individual patients and data were registered during 93 paracetamol infusions. Two infusions were excluded because the duration of infusion exceeded 5 ​min. The primary diagnoses were acute lymphocytic leukaemia, acute obstructive laryngitis, anaemia, brain haemorrhage, brain tumour, craniosynostosis, gastroenteritis, headache, hydronephrosis, meningitis, osteomyelitis, pericardial disease, pneumothorax, preterm birth, post-surgery for gastrointestinal diseases related to preterm birth, skin infection or other malignancies. The indication for paracetamol prescription was pain in all patients. Patient and treatment characteristics are presented in Table 1.

**Table 1:** Patient and treatment characteristics.

Patient characteristics	N ​= ​44 (100%)
Median age, years (range)	2.8 (0.01–17.0)
Aged <4 weeks, n ​= ​6	0.06 (0.01-0-08)
Aged 4 weeks to 2 years, n ​= ​14	0.71 (0.18–1.39)
Aged >2 to ​< ​12 years, n ​= ​16	5.56 (2.19–11.7)
Aged 12 to ​< ​18 years, n ​= ​8	15.1 (12.3–17.0)
Sex, n (%)	
Females	23 (52)
Males	21 (48)
Diagnosis, n (%)	

**Treatment characteristics**	N ​= ​44 (100%)

Indication for paracetamol	
Pain	N ​= ​44 (100%)
Catheter type	
PVC	22 (50)
CVC	22 (50)
Pain scale	
VAS	11 (25)
FLACC	22 (50)
Comfort behaviour	3 (19)
Comfort neo	8 (7)
Median infusion time in minutes:seconds (range) for individual patients (n ​= ​44)	3:00 (0:40 to 5:00)
Median infusion time in minutes:seconds (range) for all infusions (n ​= ​93)	3:00 (0:40 to 5:00)
Median paracetamol dose in mg/kg (range)	11.8 (7.5–21.0)

Abbreviations: CVC indicates Central Venous Catheter; FLACC, Face, Legs, Activity, Cry, Consolability; PVC, Peripheral Venous Catheter; VAS, Visual Analogue Scale.

### 3.1 Change in pain scores and objective signs of inflammation

**Fig. 1:**
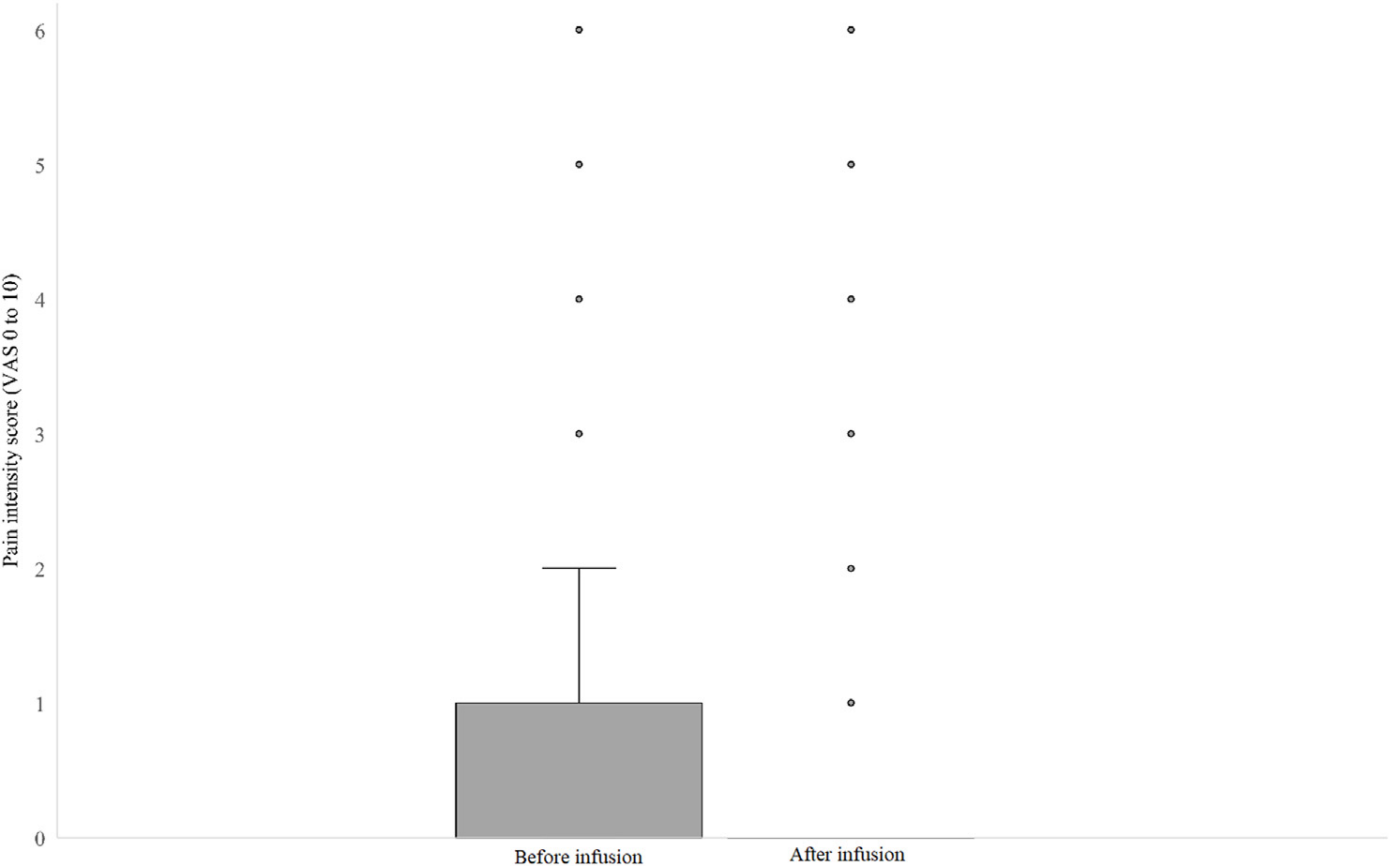
Pain intensity scores before and after paracetamol infusion: data of individual patients.

Across the 93 infusions, the mean pain score decreased, comparing pain scores before and after infusions (mean difference −0.26, 95% confidence interval (CI) −0.45 to −0.07, *P* ​= ​0.007, Table 2). In the first infusion in the 44 individual patients, there was no change in the mean pain score, comparing pain scores before and after infusions (mean difference −0.25, 95% CI -0.56 to 0.06, *P* ​= ​0.11, Table 1 and Fig. 1). In 27 out of 93 infusions using COMFORT scales, the total COMFORT score decreased, comparing total COMFORT scores before and after infusions (mean difference −1.44, 95% CI -2.77 to – 0.12, *P ​=* ​0.03). There were no signs of inflammation related to paracetamol infusions when administered via PVCs. In one patient, there was a report of pain when the infusion was aimed to be administered during less than 5 ​min, and the pain disappeared when the infusion time was changed to 10 ​min. The same patient had a previous infusion of less than 5 ​min with no report of pain.

**Table 2:** Mean change in pain score before and after paracetamol infusion: data of individual patients and of total infusions.

	Mean difference (95% CI)	*P* value
Pain scores for individual patients^a^ (n ​= ​44)	−0.25 (−0.56 to 0.06)	0.11
Pain scores for all infusions^a^ (n ​= ​93)	−0.26 (−0.45 to −0.07)	0.007

Abbreviations: CI indicates Confidence Interval.

a For patients using the COMFORT neo or behaviour scales, the VAS pain score of 0–10 is reported.

## 4 Discussion

With this safety study, we demonstrated that rapid paracetamol infusions were well-tolerated in patients below the age of 18 years. Paracetamol was successfully infused with a median infusion time of 3 ​min without inducing pain or objective signs of local inflammatory reactions, as previously shown in adults. Needleman et al. reviewed 100 medical charts of adult patients who received paracetamol with a median infusion time of 3:41 ​min, and they found only one patient who reported pain during the infusion (Needleman, 2013). In our study, one patient reported pain related to infusion, which represents 1% of the infusions. The temporary pain was registered during an infusion via a PVC, and the patient previously had a well-tolerated infusion via the same PVC. The cause of pain could multifactorial, e.g., the rapid infusion, displacement of the PVC, or anxiety about the procedure, but since a similar infusion was previously administered without pain release, it is less probably that the infusion itself released the pain.

In general, IV infusions of solutions with unphysiological osmolarities generate a high incidence of pain, whereas solutions with physiochemical properties close to plasma can be infused without causing pain (Klement and Arndt, 1991). According to the Summary of Product Characteristics of IV paracetamol, this is iso-osmotic with a pH value range of 5.0–7.0, close to the values of plasma. The recommended duration of infusion of 15 ​min is based on the experience with infusion-related pain caused by propacetamol and is neither supported by theoretical or clinical evidence.

Numerous and time-consuming infusions contribute to prolonged hours spent in inactive situations, especially for patients whose recovery depends on these infusions, e.g., in paediatric oncology. Further, health care professionals spend time with the patient during the longer infusions, which makes their work less flexible and efficient. A reduction in the duration of four daily paracetamol infusions from 1 ​h per day to 20 ​min per day may improve both the patient experience and quality of work for health care professionals.

The strengths of this study are the prospective registration of pain intensity before and after paracetamol administration in a real-world setting. The patients represented a variety of diagnoses and ages, which make the results generalisable to patients at paediatric and neonatal departments. The study is limited by the relatively small sample size, the observational safety study design without a reference group, no assessment of vital parameters during the infusions, and no registration of concomitant medication or potential drug–drug interactions.

## 5 Conclusion

Paracetamol can be administered as a rapid IV infusion in paediatric patients without causing pain or signs of inflammation. The results may contribute to more cost-effective pain control by reducing the time of inactivity for patients and health care professionals.

## Disclosure of funding support

This research did not receive any specific grant from funding agencies in the public, commercial, or not-for-profit sectors.

## Authorship statement

All persons who meet authorship criteria are listed as authors, and all authors certify that they have participated sufficiently in the work to take public responsibility for the content, including participation in the concept, design, analysis, writing, or revision of the manuscript. Furthermore, each author certifies that this material or similar material has not been and will not be submitted to or published in any other publication before its appearance in Current Research in Pharmacology and Drug Discovery.

## CRediT authorship contribution statement

**Astrid Eliasen:** Conceptualization, Methodology, Formal analysis, Data curation, Writing – original draft, Writing – review & editing, Visualization. **Sigrid Otnes:** Conceptualization, Methodology, Investigation, Resources, Writing – review & editing. **Merete Matz:** Conceptualization, Methodology, Investigation, Resources, Writing – review & editing. **Lise Aunsholt:** Conceptualization, Investigation, Resources, Writing – review & editing, Supervision. **René Mathiasen:** Conceptualization, Methodology, Writing – review & editing, Supervision.

## Declaration of competing interest

None.

## References

[bib1] Depré M., van Hecken A., Verbesselt R., Tjandra-Maga T.B., Gerin M., de Schepper P.J. (1992). Tolerance and pharmacokinetics of propacetamol, a paracetamol formulation for intravenous use. Fundam. Clin. Pharmacol..

[bib2] Flouvat B., Leneveu A., Fitoussi S., Delhotal-Landes B., Gendron A. (2004). Bioequivalence study comparing a new paracetamol solution for injection and propacetamol after single intravenous infusion in healthy subjects. Int. J. Clin. Pharmacol. Ther..

[bib3] Harris P.A., Taylor R., Minor B.L., Elliott V., Fernandez M., O'Neal L., McLeod L., Delacqua G., Delacqua F., Kirby J., Duda S.N., REDCap Consortium (2019). The REDCap consortium: building an international community of software platform partners. J. Biomed. Inf..

[bib4] Klement W., Arndt J.O. (1991). Pain on i.v. injection of some anaesthetic agents is evoked by the unphysiological osmolality or pH of their formulations. Br. J. Anaesth..

[bib5] Meremikwu M.M., Oyo-Ita A. (2002). Paracetamol versus placebo or physical methods for treating fever in children. Cochrane Database Syst. Rev..

[bib6] Needleman S.M. (2013). Safety of rapid intravenous infusion of acetaminophen. Bayl. Univ. Med. Cent. Proc..

[bib7] Perrott D.A., Piira T., Goodenough B., Champion G.D. (2004). Efficacy and safety of acetaminophen vs ibuprofen for treating children's pain or fever: a meta-analysis. Arch. Pediatr. Adolesc. Med..

[bib8] Sharma C.V., Mehta V. (2014). Paracetamol: mechanisms and updates. Cont. Educ. Anaesth. Crit. Care Pain.

[bib9] Sinatra R.S., Jahr J.S., Reynolds L.W., Viscusi E.R., Groudine S.B., Payen-Champenois C. (2005). Efficacy and safety of single and repeated administration of 1 gram intravenous acetaminophen injection (paracetamol) for pain management after major orthopedic surgery. Anesthesiology.

[bib10] Tan E., Braithwaite I., McKinlay C.J.D., Dalziel S.R. (2020). Comparison of acetaminophen (paracetamol) with ibuprofen for treatment of fever or pain in children younger than 2 years: a systematic review and meta-analysis. JAMA Netw. Open.

